# Bilateral Posterior Native Hip Dislocations after Fall from Standing

**DOI:** 10.5811/cpcem.2017.8.35161

**Published:** 2017-10-06

**Authors:** Jane Xiao, Joseph A. Hamera, Christopher H. Hutchinson, David A. Berger

**Affiliations:** * Oakland University William Beaumont School of Medicine, Department of Emergency Medicine, Rochester, Michigan; † Beaumont Health System, Department of Emergency Medicine, Michigan

## Abstract

We present a case of bilateral posterior native hip dislocations after a fall from standing. This exceedingly rare diagnosis is classically associated with younger patients whose bones are strong enough to dislocate rather than fracture in the setting of a high-momentum collision. We present an unusual case of an 88-year-old male with native hips who sustained a low-energy collision after falling from standing and was found to have bilateral posterior hip dislocations without associated pelvis or femur fractures.

## INTRODUCTION

Ninety percent of native hip dislocations are posterior dislocations.^1^ They are classically associated with motor vehicle collisions, which cause the vast majority of traumatic posterior hip dislocations.^2^ In these situations, patients often sustain multiple traumatic injuries due to the high momentum required to dislodge the femoral head. Both hips are held in flexion and adduction with axial loading to the femur, usually via a flexed knee striking a dashboard. About 400 newtons of force are required to cause hip joint separation.^3^ Due to the large force required to cause a native hip dislocation, there is a 95% incidence of injury to other areas of the body in these patients, especially injuries to the knee.^4^ Other risk factors include but are not limited to prior hip dislocation, hip prosthetics, joint laxity from underlying medical condition such as Down syndrome and Ehlers-Danlos syndrome. Children can get hip dislocations from smaller forces, such as a fall from standing, due to immature development of the joint.

A posteriorly dislocated hip usually presents foreshortened, flexed, internally rotated, and adducted. The greater trochanter and buttock may be more prominent to visualization and palpation. An anterior-posterior pelvis and lateral radiograph may easily confirm a hip dislocation. Obtaining radiographs prior to attempting reduction is important in order to identify associated fractures, which may make reduction more difficult. Fractures of the femoral neck and associated lower limb are relative contraindications to attempting closed reduction in the emergency department (ED).

Simultaneous bilateral traumatic hip dislocations is a true emergency. Prompt reduction within six hours is important to prevent complications including osteonecrosis, as well as the development of scar tissue and joint instability, which may impede joint reduction.^5^–^7^ Although several closed reduction techniques have been described, a popular method used in the ED is the Allis reduction maneuver. Indications for surgical management include multiple failed attempts at closed reduction, intraarticular bony fragments causing incongruous reduction, neurovascular compromise, dislocation-fracture combination injuries, and inability to tolerate bedside anesthesia.

Common complications of posterior dislocations include avascular necrosis and traumatic arthritis. Avascular necrosis, caused by disruption to the circumflex femoral artery, is the dreaded complication of hip dislocation and occurs in 10% of cases.^5^ Other complications include injury to sciatic nerve, specifically the peroneal branch that is stretched over the displaced femoral head, potentially causing transient or permanent nerve injury.^8^–^9^ Prognosis is determined by several factors including time to reduction, overall trauma severity, age, comorbidities and frailty.^10^ Patients are often allowed to weight bear as tolerated afterwards, with close orthopedic and radiologic follow-up.

## CASE REPORT

An 88-year-old Caucasian male presented to our ED by ambulance after being found unresponsive on the floor of his home by family. On arrival he was pale and mottled, with a Glasgow Coma Scale of 3. He was normothermic, tachycardic in sinus rhythm, hypertensive, and severely hypoxic. Traumatic injuries on exam were significant for large anterior chest wall contusion, right leg laceration, and inwardly rotated legs of equal length with symmetric hips. Peripheral pulses were 1+ palpable. The patient was intubated in upright positioning due to oxygen desaturations while lying flat. An orogastric tube drained coffee-ground fluid and a urethral catheter initial efflux was clear yellow and then transitioned to gross hematuria. The patient’s initial labs were significant for influenza A, acute kidney injury, ischemic hepatitis, rhabdomyolysis, lactic acidemia, non ST-segment elevation myocardial infarction, and a negative comprehensive drug screen. His family arrived later and provided additional history. He had been a healthy, independent octogenarian who played tennis weekly, had no medical problems, and took no prescription medications. In the week prior to the incident, the patient exhibited flu-like symptoms but sounded well on the phone one day prior to presentation. When they found him unresponsive, he was not found near stairs.

**Image 1:**
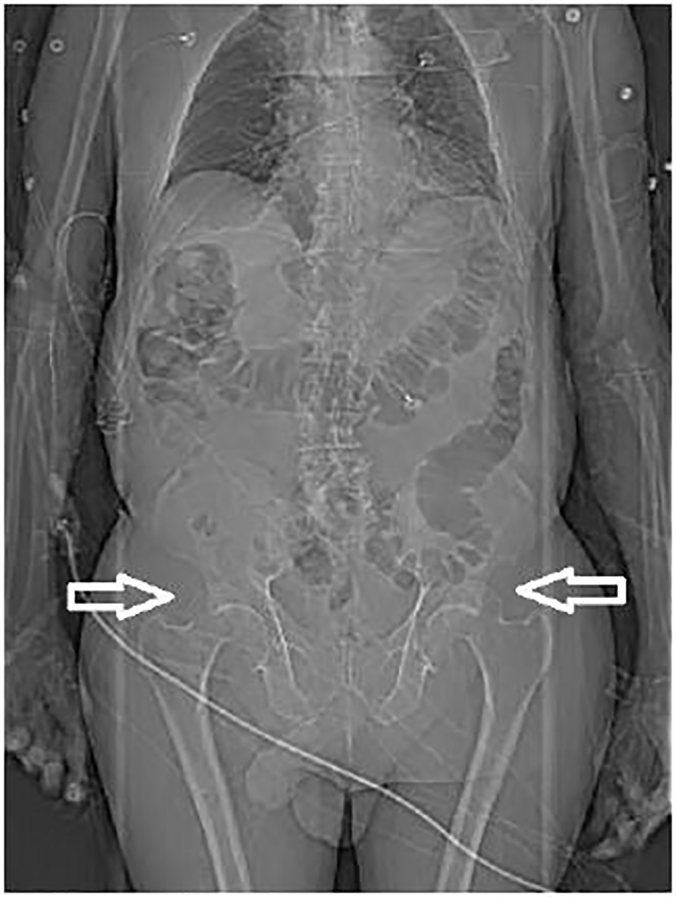
Computed tomography scout image demonstrating bilateral posterior hip dislocations (arrows).

**Image 2:**
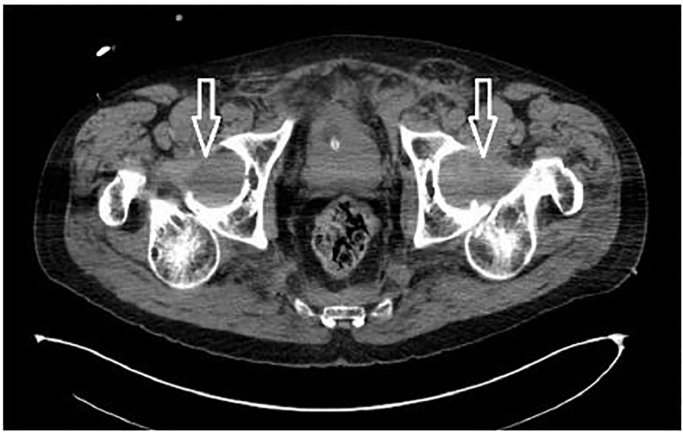
Computed tomography axial image demonstrating bilateral posterior hip dislocations (arrows).

**Image 3:**
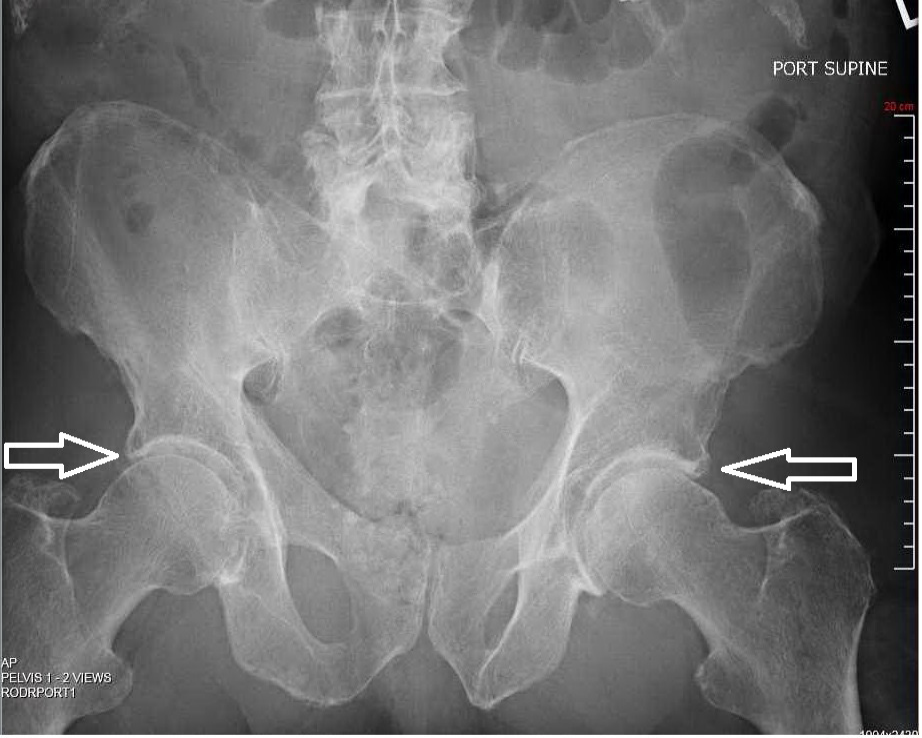
Anterior-posterior pelvic radiograph demonstrating the hips after reduction (arrows).

Computed tomography revealed bilateral posterior hip dislocations with both femoral heads superior and posterior to the acetabulum (Image 1 and Image 2). There were no pelvic fractures. Bilateral closed hip reduction was performed at the bedside using the Allis reduction maneuver. With the hip stabilized by an assistant, traction was applied to the femur with the knee in flexion, as the hip was slowly flexed to 90 degrees. An obvious “clunk” occurred as the femoral head slid back into the acetabulum. The hip was then slowly extended maintaining traction and the leg positioned in abduction and external rotation while post-reduction films were obtained. There was concern for right hip instability because the patient required multiple right hip reductions, including axial traction as described above, and modified Allis maneuver without knee flexion. The left hip was reduced easily with axial traction. Both legs were placed in knee immobilizers and positioned in hip abduction and flexion. Reduction was confirmed with AP pelvis radiograph (Image 3). Dorsalis pedis and posterior tibial pulses were palpable before and after the reduction. The patient was transferred to the intensive care unit (ICU).

**CPC-EM Capsule**

What do we already know about this clinical entity?

Posterior hip dislocations classically occur with high-momentum injuries in motor vehicle collisions or a fall from a great height. They present with an inwardly rotated and shortened leg.

What makes this presentation of disease reportable?

We present a rare case of bilateral posterior hip dislocations in native hip joints after a fall from standing.

What is the major learning point?

A thorough physical exam is crucial to securing this rare but important diagnosis in unresponsive patients for timely reduction in the emergency department.

How might this improve emergency medicine practice?

In unstable and unresponsive patients, the physical exam is an important tool for the emergency physician to identify life- or limb-threatening diagnoses.

The patient was awake and following commands while intubated on ICU day 1, and was extubated on ICU day 10. His only neurologic deficit was mild difficulty with concentration. On follow-up, patient recalled that he was standing on a level surface and reaching for a glass of water when he lost balance and fell with his legs “doing the splits.” He denied head trauma or loss of consciousness with his fall. He was unable to call for help despite dragging himself across the floor, and he lay on the ground for approximately 30 hours. He had never sustained prior hip injury or dislocation. On ICU day 9, orthopedic surgery performed an exam under anesthesia and found no joint instability. Later, he developed decreased sensation in the right leg attributed to sciatic neuropraxia. His hospital course was complicated by multisystem organ failure requiring initiation of dialysis, possible aspiration pneumonia, atrial fibrillation with rapid ventricular rate, metabolic encephalopathy, and hematochezia with acute blood loss anemia. He was eventually transferred to the inpatient hospice unit and expired on hospital day 23.

## DISCUSSION

We present an unusual case of bilateral posterior hip dislocations with an atypical mechanism of low- momentum collision in native hip joints. Bilateral posterior hip dislocations are rare; there are more case reports of hip dislocations with one anterior and the other posterior compared to bilateral posterior hip dislocations.^11^^–22^ The majority were associated with classic high-momentum injuries such as a motor vehicle collision, fall from a great height, or inherent joint instability. Clinically a posterior hip dislocation presents with a foreshortened, adducted and internally rotated leg, most evident when compared to the contralateral leg.

In intubated patients with bilateral symmetric hip dislocations, the physician does not have the clue of leg asymmetry or a patient-provided history. In this case, it was initially mistakenly attributed to chronic contractures often seen in elderly bedridden patients. However, leg contractures usually present with the legs externally rotated. In addition, this patient’s physical exam findings of significant chest contusions and leg lacerations should have prompted a higher level of clinical suspicion of other traumatic injuries despite the limited history available at the time of initial evaluation. Although this case report represents an outlier in the grand scheme of hip dislocations, it is an interesting and atypical presentation of a classic emergency medicine diagnosis.

## CONCLUSION

Although classically associated with high-momentum collisions, we present a rare case of bilateral posterior native hip dislocations after a low-momentum injury. This diagnosis was not considered after initial physical examination because the patient was unable to provide a history and had symmetric appearance of the lower extremities. In these cases, emergency physicians should perform a thorough physical exam in order to secure the diagnosis and perform closed reduction in a timely manner.
